# Supplementary material for: Versatile tunable optical injection of chiral polarized Weyl fermions in a magnetic Weyl semimetal Co3Sn2S2
Source: Natl Sci Rev. 2025 Sep 27;12(12):nwaf402. doi: 10.1093/nsr/nwaf402 (PMC12704104; doi:10.1093/nsr/nwaf402)
Supplement: nwaf402_Supplemental_File [file nwaf402_supplemental_file.docx]

**Versatile tunable optical injection of chiral polarized weyl fermions in** **a magnetic weyl semimetal Co_3_Sn_2_S_2_**

Zipu Fan, Junchao Ma, Jinying Yang, Yan Sun, Zhuocheng Lu, Shuxia Chen, Delang Liang, Dehong Yang, Chang Xu, Qinsheng Wang, Anlian Pan, Ji Feng, Enke Liu^†^, JinLuo Cheng^†^, Dong Sun^†^

***Supplementary Material***

**Table of Contents:**

**I. Fourier transform of the *θ_λ/4_*-dependent photocurrent**

**II. Measurement of *I_Λ_* via electrodes along the *x*-axis**

**III. Measurement of *I_Λ_* at the interfaces between the electrodes and Co_3_Sn_2_S_2_**

**IV. Temperature dependence of *I_Λ_***

**V. Absence of *I_Λ_* under near-infrared excitations**

**VI. Confirmation of the sign switch of *I_Λ_* through the SPCM**

**VII. Confirmation of the sign switch of *I_Λ_* on an additional device**

**VIII. Bias dependence of *I_Λ_* under 10.6-μm excitation**

**IX. Optical matrix element in the vicinity of the Weyl point**

**X. Potential contributions to the third-order nonlinear photocurrent**

**XI. Numerical calculations of magnetic circular dichroism**

**XII. Tunability of *I_Λ_* measured at the central area of the sample**

**I.** **Fourier transform of the *θ_λ/4_*-dependent photocurrent**

In this section, we extract the components of the photocurrent with different periodicities through the Fourier transform. The Fourier transform of the *θ_λ/4_*-dependent photocurrent measured under -*M* magnetization, as presented in Fig. 2b of the maintext, is shown in Fig. S1a. Five peaks are observed at angular frequencies of 0, 1/180°, 1/90°, 1/45° and 1/30°. The 180°-period component corresponds to the light chirality-dependent photocurrent. To illustrate this, the photocurrent components of different periodicities are plotted separately in Fig. S1b. The 180°-period component clearly corresponds to the difference in the photocurrent between LCP and RCP excitations. Therefore, the light chirality-dependent photocurrent is identified as the 180°-period component after Fourier transform of the *θ_λ/4_*-dependent photocurrent.

Next, we briefly discuss the physical implications of the 45°-period and 30°-period components. Two fitting curves of the *θ_λ/4_*-dependent photocurrent with and without the 45°-period and 30°-period components are plotted in Fig. S1c. The 45°-period and 30°-period components clearly directly correspond to the sharp peaks in the photocurrent at quarter-wave plate (QWP) angles of 45° and 135°, respectively. A straightforward explanation for the higher frequency terms is the higher-order nonlinear response, which matches the angular periodicity observed in the experiment. However, higher-order nonlinear effects typically result in nonlinear power dependence and can potentially influence the power dependence of the 180°-period component. In our experiments, we do not observe a significant nonlinear power dependence in the 180°-period component (Fig. 2d of the main text), indicating that the higher frequency terms may have contributions from other unknown effects. Although the exact mechanisms behind the higher-order nonlinear effect remain unknown, we note that the detailed mechanisms are not relevant to the analysis of our work and thus do not affect any conclusions of this work. A comprehensive understanding of the higher frequency terms would require further investigation, which is not the focus of this work.

**
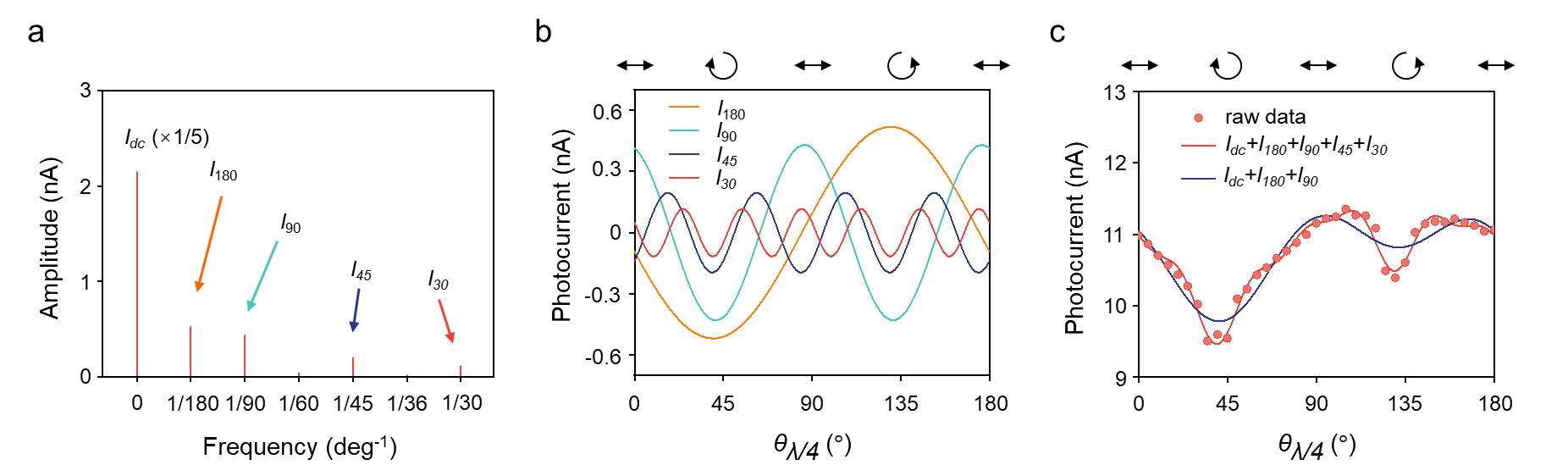
**

**Figure S1****.** Fourier transform of the light chirality-dependent photocurrent. (a) Fourier transform of the light chirality-dependent photocurrent measured for device 1. The direct current (DC) component is multiplied by 1/5. (b) Photocurrent components of different periodicities after the Fourier transform. (c) Fitting curves with and without the additional 45°-period and 30°-period components.

**II. Measurement of *I_Λ_* via electrodes along the *x*-axis**

To study the relationship between the light chirality-dependent photocurrent (*I_Λ_*) and the orientation between the crystallographic direction and the electrodes, we also measured the photocurrent using electrodes along the *x*-axis. Both the spatially resolved and light chirality-dependent photocurrents were measured, as shown in Fig. S2. The light chirality-dependent photocurrent also clearly linearly depends on the external bias and can be controlled by the ferromagnetic order, which is consistent with the results obtained using electrodes along the *y*-axis, as shown in the main text. This finding indicates that the observed light chirality-dependent photocurrent is independent of the orientation between the sample and the electrodes.

Additionally, we note that the slopes in Fig. S2b are asymmetric under *+M* and *-M* magnetizations. We attribute this to the influence of the hotspot, which is partially covered by the large spot at 4 μm during the measurement (see the negative response peak in Fig. S2a). As described in our main text, when bias-dependent experiments were conducted, we focused the light spot on the center of the sample to minimize the influence of the response at the electrode‒sample interface. However, the asymmetric results due to the presence of hotspots do not affect our conclusion that *I_Λ_* has opposite signs under *+M* and *-M* magnetizations.

**
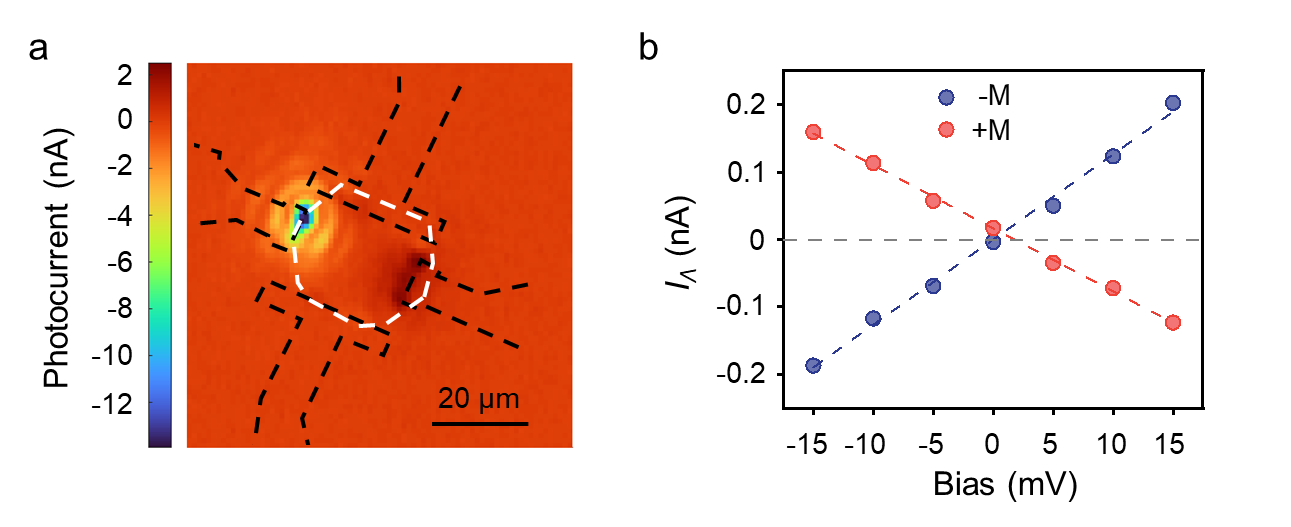
**

**Figure S2.** Light chirality-dependent photocurrent measurement using a pair of electrodes along the *x*-axis. (a) Scanning photocurrent microscopy using a pair of electrodes along the *x*-axis. (b) External bias dependence of the light chirality-dependent photocurrent under different magnetizations. The dashed line is the linear fit of the data.

**III. Measurement of *I_Λ_* at the interfaces between the electrodes and Co_3_Sn_2_S_2_**

In this section, we performed measurements at the interface between the electrodes and Co_3_Sn_2_S_2_ on an additional device (device 2), as shown in Fig. S3a. As illustrated in Fig. S3b, the scanning photocurrent microscopy (SPCM) image shows that the photocurrent response is limited to the interface area with the electrodes and has opposite signs at the two opposite electrodes. The light chirality-dependent photocurrent is then measured at the two edges of the electrodes, which are denoted as p1 and p2. As shown in Fig. S3c, the magnitude of the photocurrent is different under LCP and RCP excitations, which indicates the existence of a light chirality-dependent photocurrent. *I_Λ_* has opposite signs at the interfaces with the two electrodes because the built-in electric field switches signs at the two interfaces. Furthermore, when the magnetization direction is reversed, the sign of *I_Λ_* is also reversed. The sign of the total photocurrent remains unchanged, as the total response is dominated by a light chirality-independent photoresponse.

**
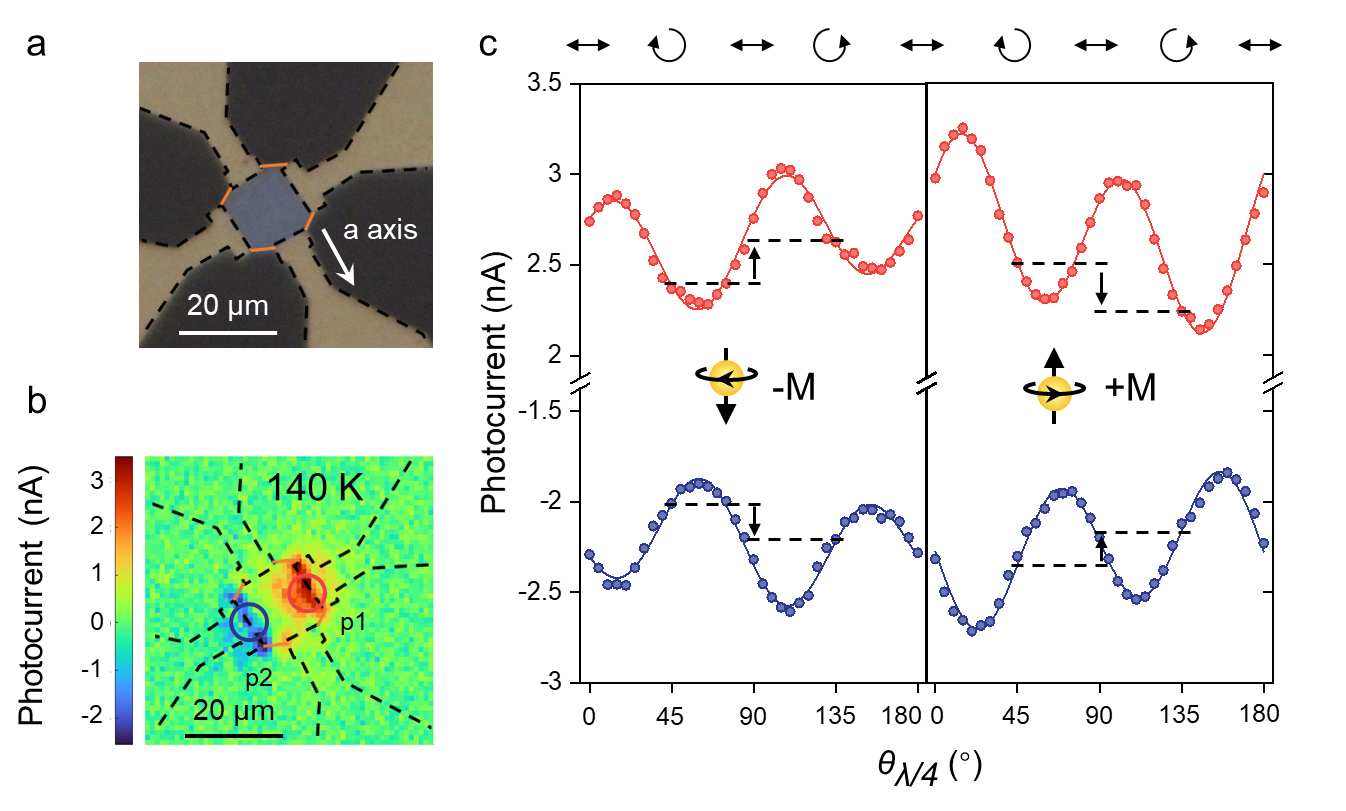
**

**Figure S3.** Photocurrent measurements at the interface between the electrodes and Co_3_Sn_2_S_2_ using a pair of electrodes along the *y*-axis. (a) Optical microscopy image of Co_3_Sn_2_S_2_ device 2. (b) Scanning photocurrent microscopy image of device 2 using a pair of electrodes along the *y*-axis. The red and blue circles denote the positions (p1 and p2, respectively) where the light chirality-dependent photocurrent is measured. (c) Light chirality-dependent photocurrent results after cooling to 140 K with -*M* (left panel) and +*M* (right panel) magnetizations. The red and blue curves correspond to the measurement locations marked by red and blue circles in (b).

We also repeat the measurement by using the electrodes along the *x*-axis. The spatially resolved and polarization-dependent photocurrent responses were measured, as shown in Fig. S4. For comparison, the light chirality-dependent photocurrent component at the interface with the electrodes was extracted and is shown in Fig. S4c. Regardless of which pair of electrodes is used, the light chirality-dependent photocurrent exhibits similar characteristics: *I_Λ_* has opposite signs at the interface with the two connected electrodes and can be controlled by the ferromagnetic order.

**
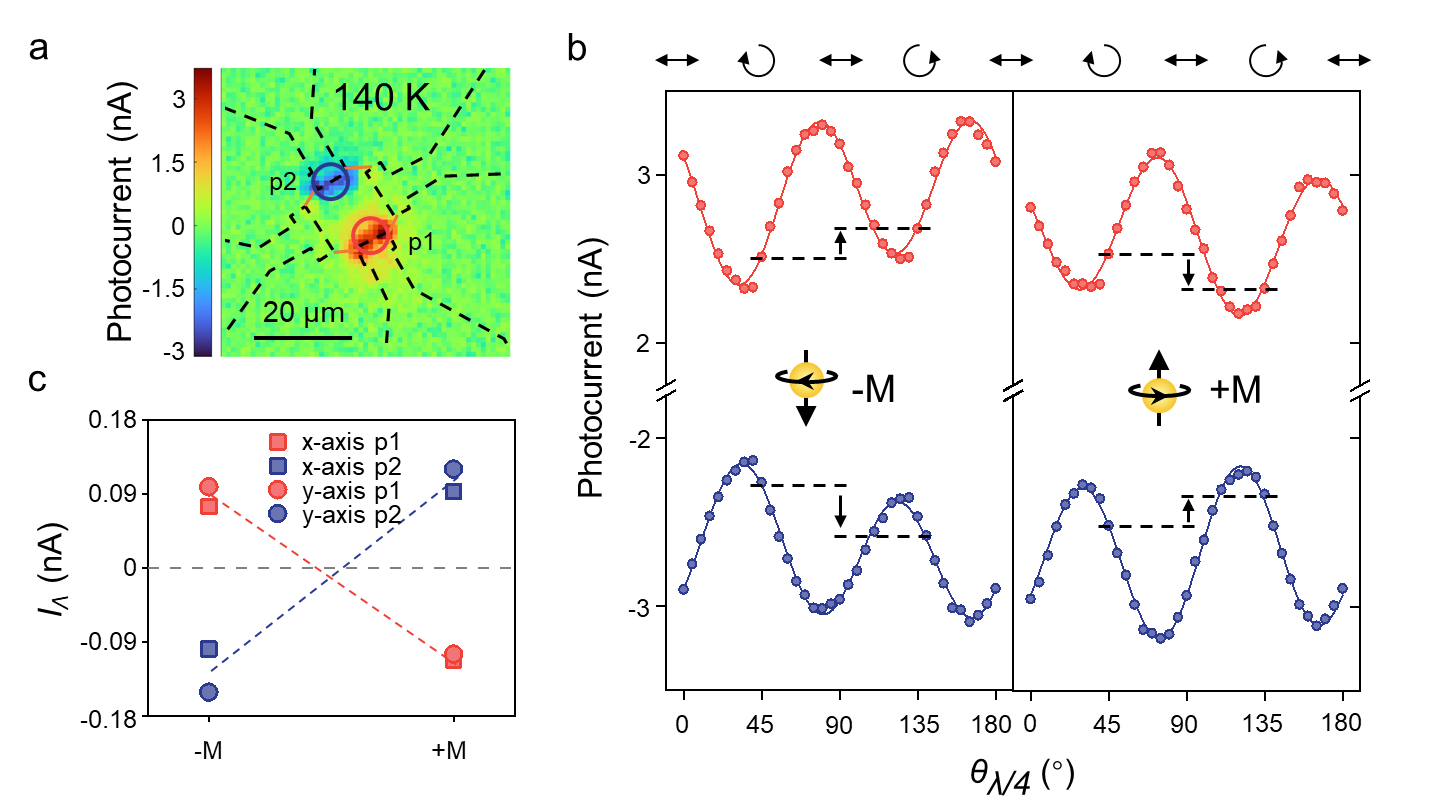
**

**Figure S4.** Photocurrent measurements at the interfaces between the electrodes and Co_3_Sn_2_S_2_ using the pair of electrodes along the *x*-axis. (a) Scanning photocurrent microscopy image of device 2 using a pair of electrodes along the *y*-axis. The red and blue circles denote the positions (p1 and p2, respectively) where the light chirality-dependent photocurrent is measured. (b) Light chirality-dependent photocurrent results after cooling to 140 K with -*M* (left panel) and +*M* (right panel) magnetizations. The red and blue curves correspond to the measurement locations marked by red and blue circles in (a). (c) Comparison of the extracted light chirality-dependent photocurrents measured using different pairs of electrodes. The square and circle represent the measurement results obtained via the pair of electrodes along the *x*-axis and the *y*-axis, respectively.

**IV. Temperature dependence of *I_Λ_***

In this section, we present the results of light chirality-dependent photocurrent (*I_Λ_*) measurements at different temperatures. The temperature dependence of *I_Λ_* measured under 4-μm excitation from 25 K to 180 K (slightly above the ferromagnetic transition temperature of Co_3_Sn_2_S_2_, 177 K) is shown in Fig. S5. The bias dependence of *I_Λ_* measured under 4-μm excitation at room temperature is shown in Fig. S6. A significant *I_Λ_* response is observed in the ferromagnetic phase (25 K–170 K), whereas it vanishes abruptly once the temperature exceeds the transition point—even by just a few kelvins (180 K). This result suggests that the formation of the Weyl phase plays a crucial role in the observation of the light chirality-dependent photocurrent *I_Λ_*.


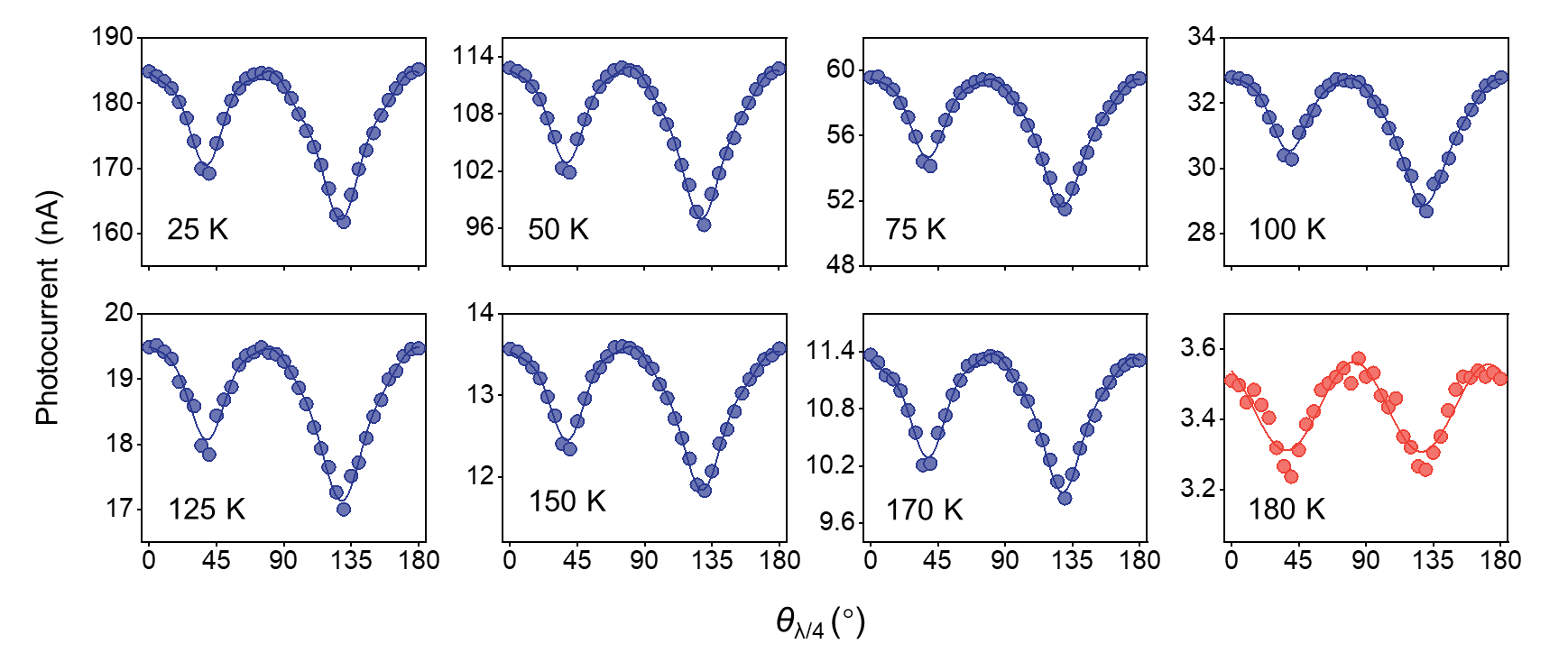


**Figure S5.** Light chirality-dependent photocurrent measured at different temperatures. The measurements were performed under 4-μm excitation with a 10-mV bias applied.

*
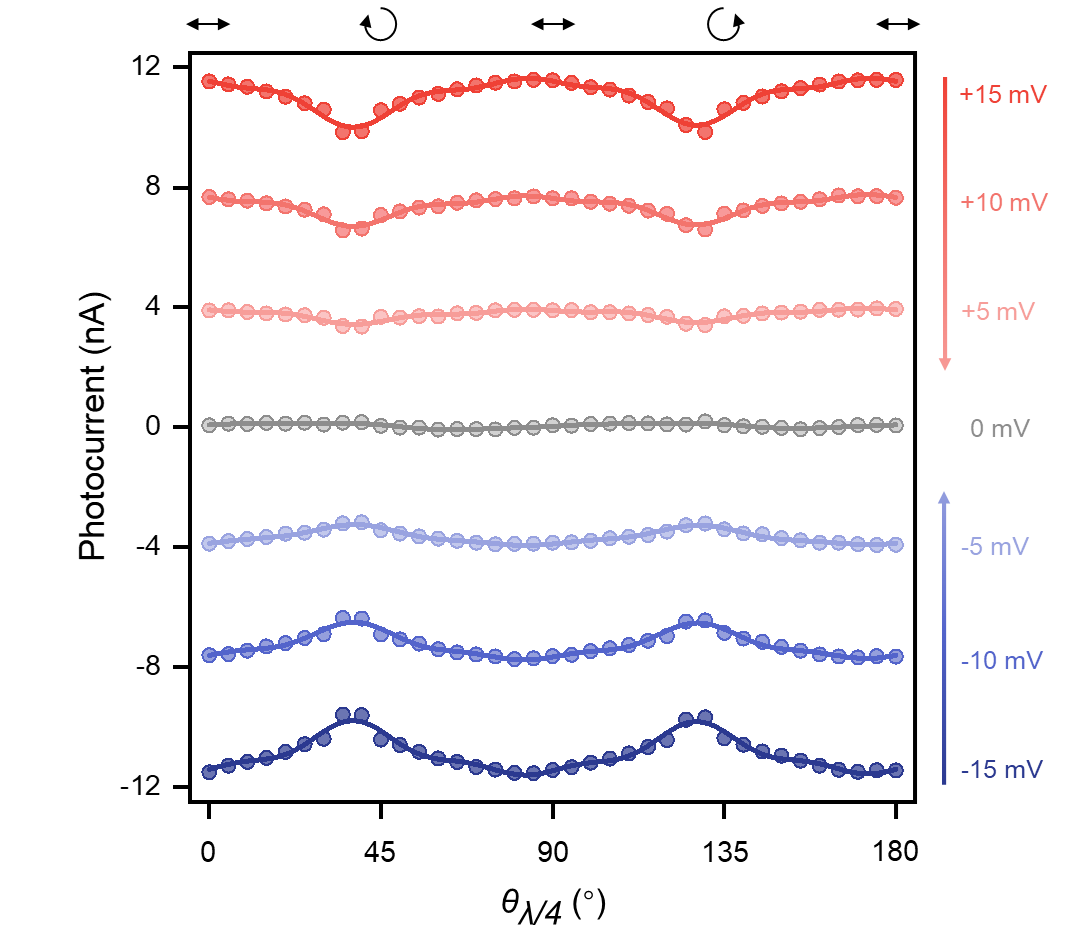
*

**Figure S6.** Light chirality-dependent photocurrent measured at room temperature under different bias voltages. The measurements were performed under 4-μm excitation.

**V. Absence of *I_Λ_* under near-infrared excitations**

In this section, we present the results of light chirality-dependent photocurrent (*I_Λ_*) measurements under near-infrared excitation. The bias dependence of the light chirality-dependent photocurrent measured under 1550-nm and 800-nm excitation is shown in Fig. S7. However, in contrast to those under mid-infrared excitation, we do not observe a clear light chirality-dependent photocurrent under 1550 nm and 800-nm excitation.


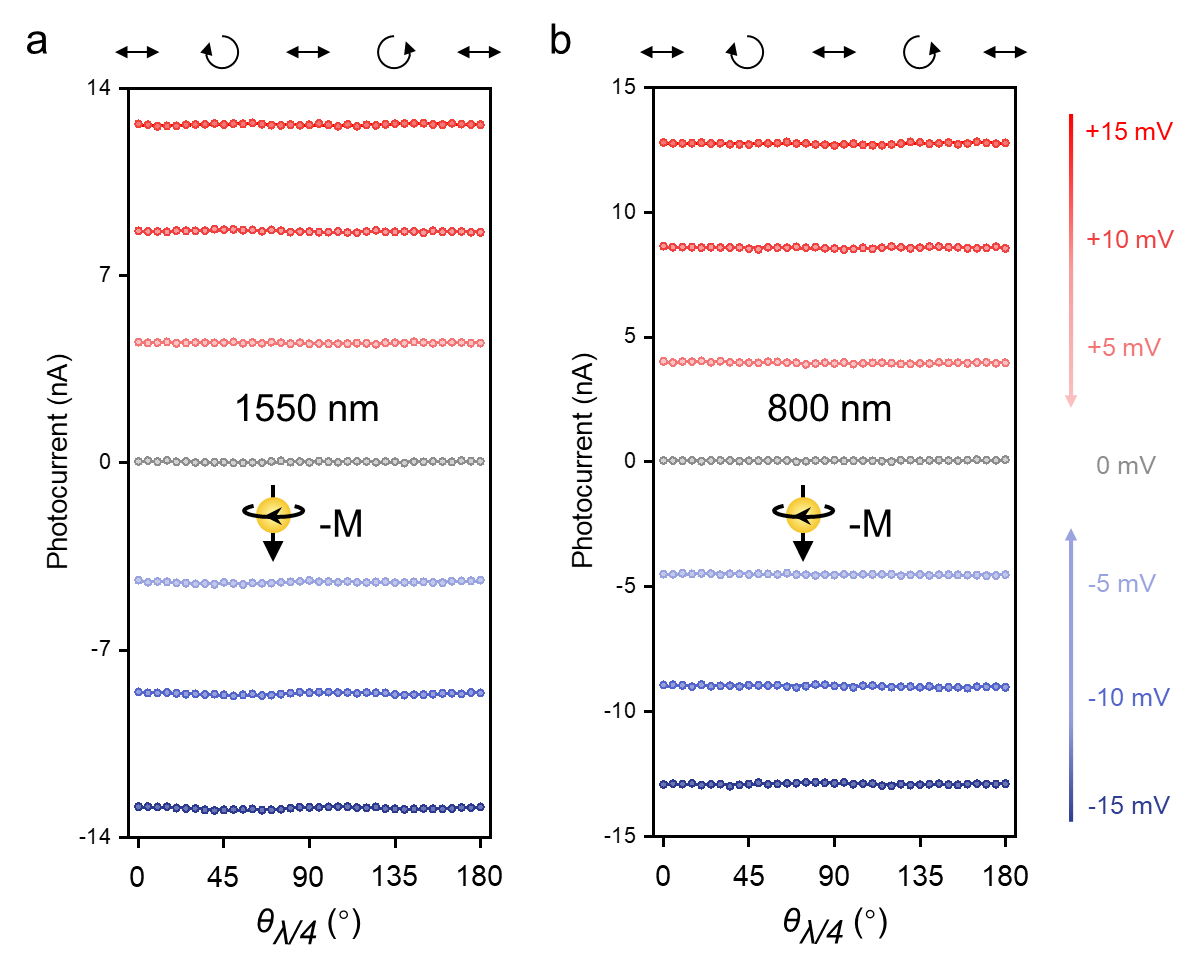


**Figure S7.** Bias dependence of the light chirality-dependent photocurrent measured under 1550-nm (a) and 800-nm excitation (b), respectively. The measurements were performed after cooling to 140 K with -*M* magnetization.

**VI. C****onfirmation of the sign switch of *I_Λ_* through the SPCM**

To verify the sign of the light chirality-dependent photocurrent (*I_Λ_*) under 4-μm and 10.6-μm excitation, we also performed SPCM measurements under LCP and RCP excitation with a 10-mV bias voltage applied. The space distribution of the light chirality-dependent photocurrent is obtained by subtracting the photocurrent measured under LCP and RCP excitations. The results under 4-μm and 10.6-μm excitation are shown in Fig. S8 and Fig. S9, respectively. Under the same magnetization direction, the signs of the light helicity-dependent photocurrent are opposite for the 4-μm and 10.6-μm excitations, which is consistent with the results shown in Fig. 3a of the main text. In Fig. S10, we also present the results with a -10-mV bias voltage applied under 4-μm excitation to confirm the sign switch of *I_Λ_* when the direction of the external electric field is changed.

**
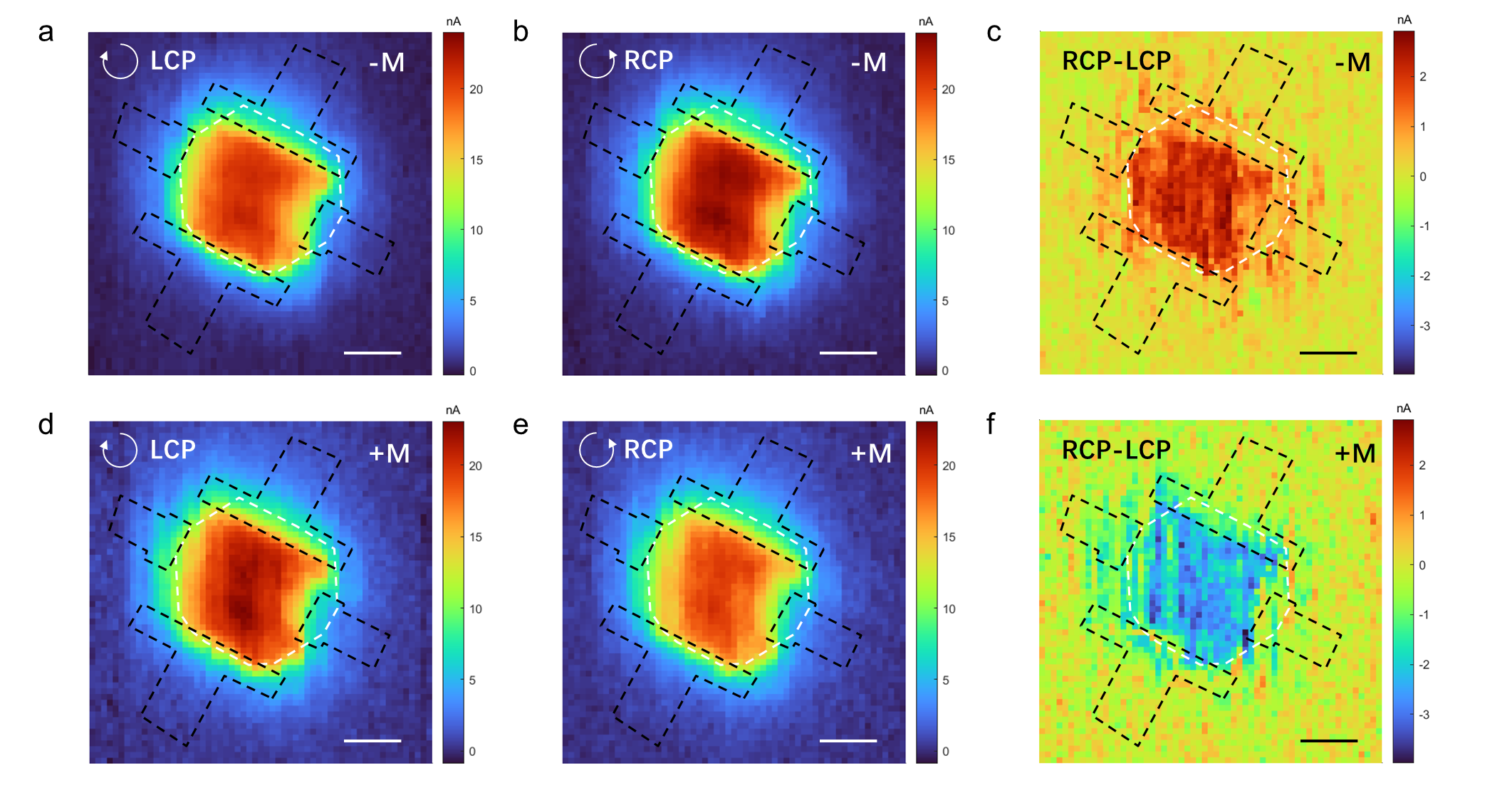
**

**Figure S8.** Scanning photocurrent microscopy images under 4-μm LCP and RCP excitations. (a), (b) SPCM images taken after cooling to 140 K with -*M* magnetization under LCP and RCP excitations, respectively. The measurements were performed with a 10-mV bias voltage applied. (c) Photocurrent difference between LCP and RCP excitations. (d)-(f) Results measured under +*M* magnetization similar to (a)-(c), respectively. All scale bars are 10 μm.


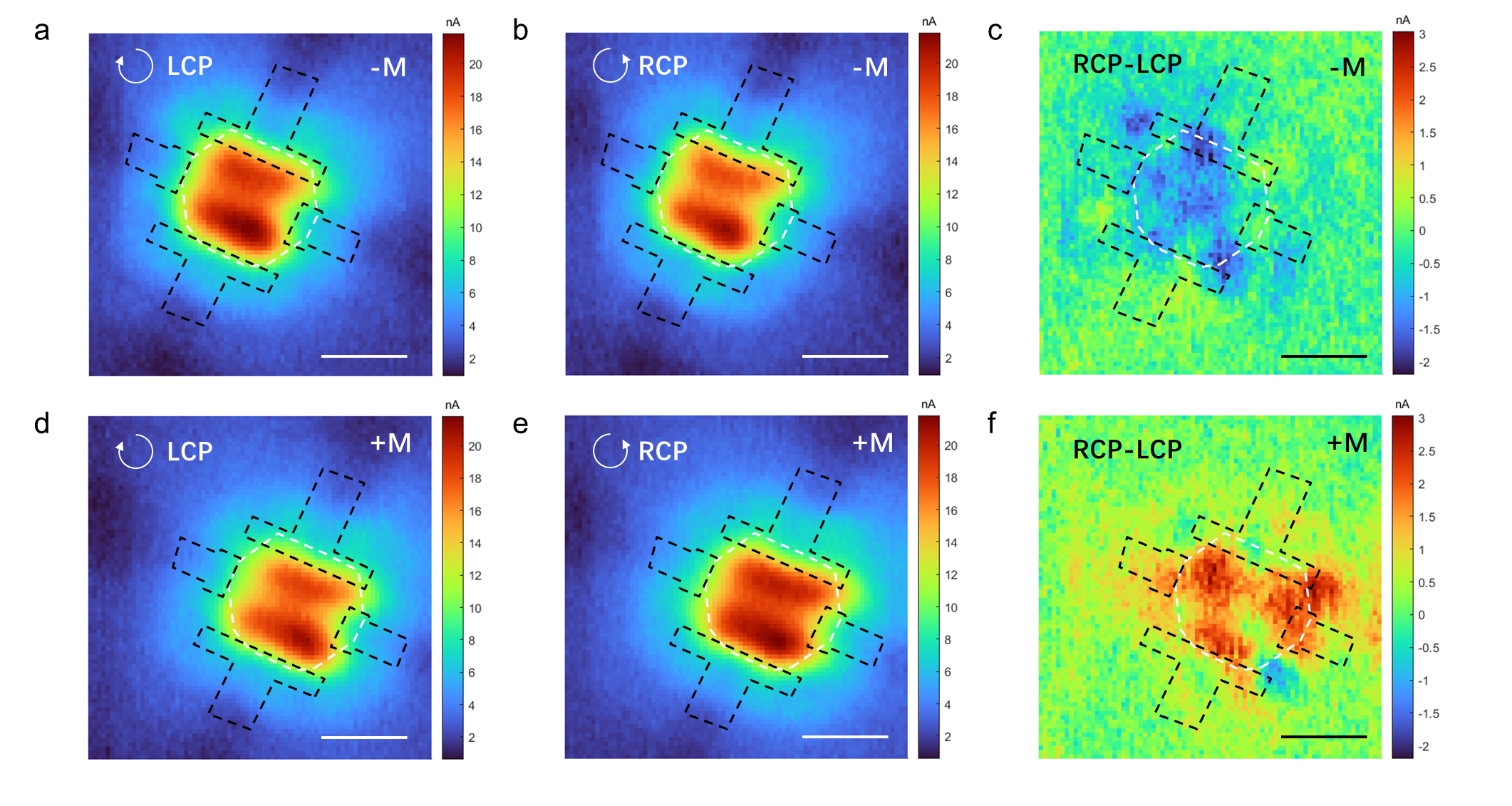


**Figure S9** Scanning photocurrent microscopy images under 10.6-μm LCP and RCP excitations. (a), (b) SPCM images taken after cooling to 140 K with -*M* magnetization under LCP and RCP excitations, respectively. The measurements were performed with a 10-mV bias voltage applied. (c) Photocurrent difference between LCP and RCP excitations. (d)-(f) Results measured under +*M* magnetization similar to (a)-(c), respectively. All scale bars are 20 μm.

**
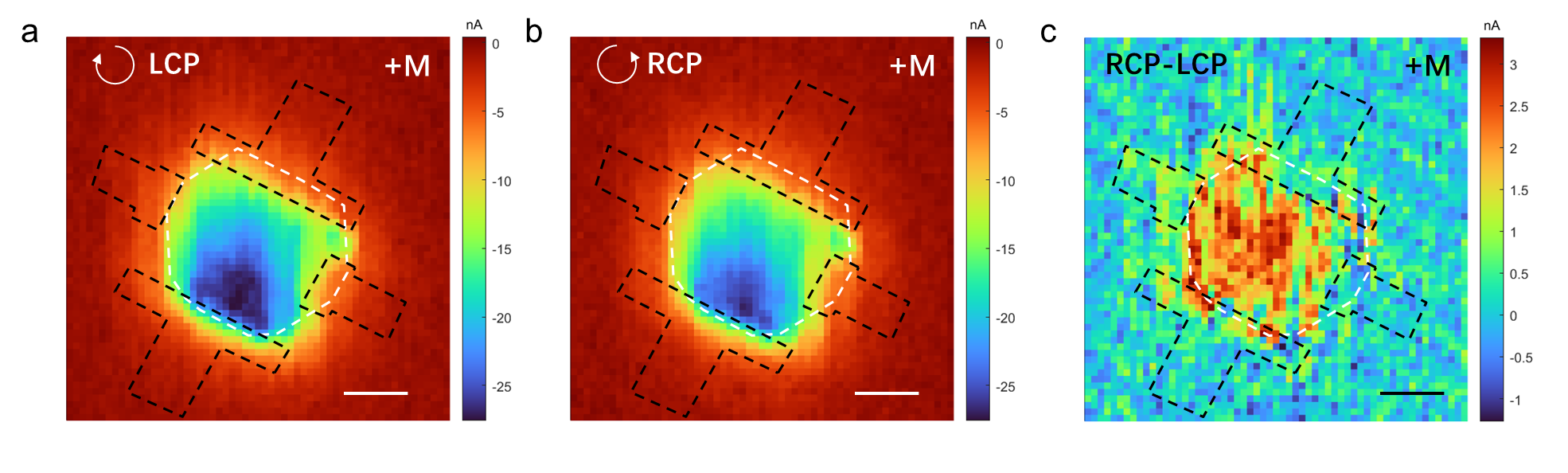
**

**Figure S10.** Scanning photocurrent microscopy images under 4-μm LCP and RCP excitations with a -10-mV bias voltage applied. (a), (b) SPCM images taken after cooling to 140 K with +*M* magnetization under LCP and RCP excitations, respectively. (c) Photocurrent difference between LCP and RCP excitations. All scale bars are 10 μm.

**VII. Confirmation of the sign switch of *I_Λ_* on an additional device**

To further confirm the sign switching of the light chirality-dependent photocurrent (*I_Λ_*) under 4-μm and 10.6-μm excitation, we performed photocurrent measurements on an additional device (device 3). As shown in Fig. S11, both the light chirality-dependent photocurrent and the SPCM results clearly reveal reversal of the light chirality-dependent photocurrent under excitation at the two wavelengths, which is consistent with the results shown in Fig. 3a of the main text.

**
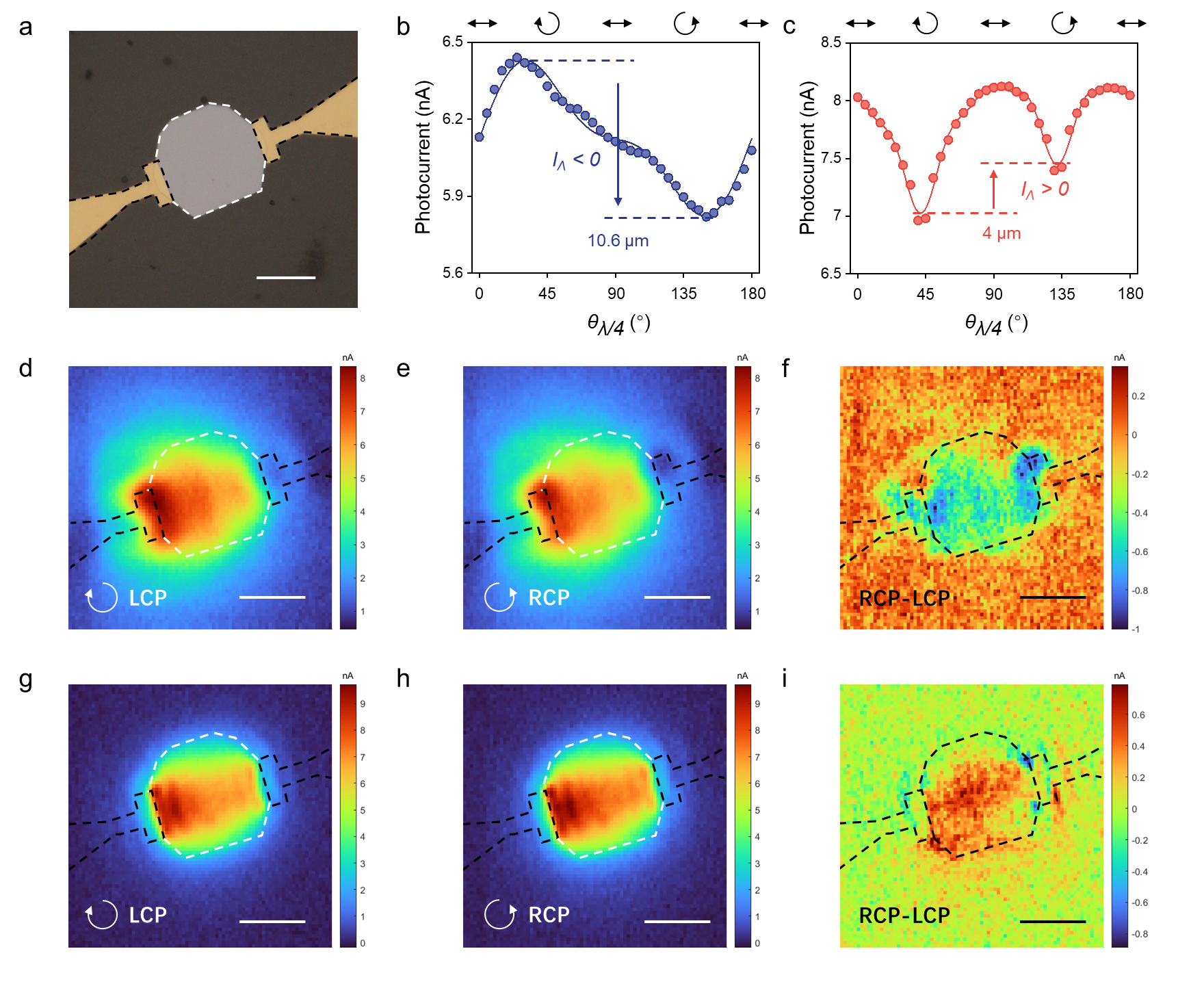
**

**Figure S11.** Photocurrent measurements of Co_3_Sn_2_S_2_ device 3 at 140 K. (a) Optical microscopy image of Co_3_Sn_2_S_2_ device 3. (b), (c) Light chirality-dependent photocurrents measured under 10.6-μm and 4-μm excitations, respectively. (d), (e) SPCM images taken under 10.6 μm LCP and RCP excitations, respectively. (f) Photocurrent difference between 10.6-μm LCP and RCP excitations. (g)-(i) Results measured under 4-μm excitation, similar to (d)-(f), respectively. All the measurements were taken under -*M* magnetization at 140 K, with a 10-mV bias applied. All scale bars are 20 μm.

In addition, we performed measurements at a lower temperature (25 K). The measurements were taken under +*M* magnetization at 25 K, with a 10-mV bias applied. As shown in Fig. S12, the key experimental feature, the opposite sign of *I_Λ_* under 4-µm and 10.6-µm excitation, remains the same as that measured at 140 K. The consistency between the experimental results at 25 K and 140 K suggests that our interpretation remains valid at a broad temperature range of 25 K--140 K.


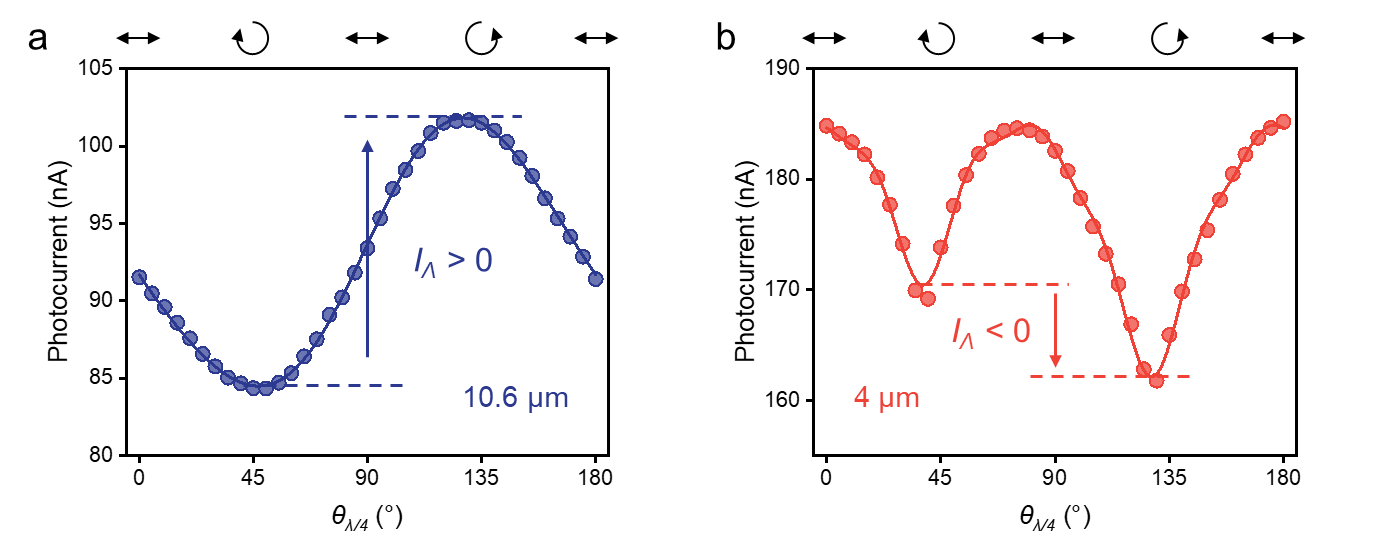


**Figure S12.** Photocurrent measurements of Co_3_Sn_2_S_2_ device 3 at 25 K. (a), (b) Light chirality-dependent photocurrents measured under 10.6-μm and 4-μm excitations, respectively. All the measurements were taken under +*M* magnetization at 25 K, with a 10-mV bias applied.

**VIII. Bias dependence of *I_Λ_* under 10.6-μm excitation**

The bias dependence of the light chirality-dependent photocurrent (*I_Λ_*) measured under 10.6-μm excitation is shown in Fig. S13. The measurements were performed on Co_3_Sn_2_S_2_ device 1 after cooling to 140 K with -*M* magnetization.


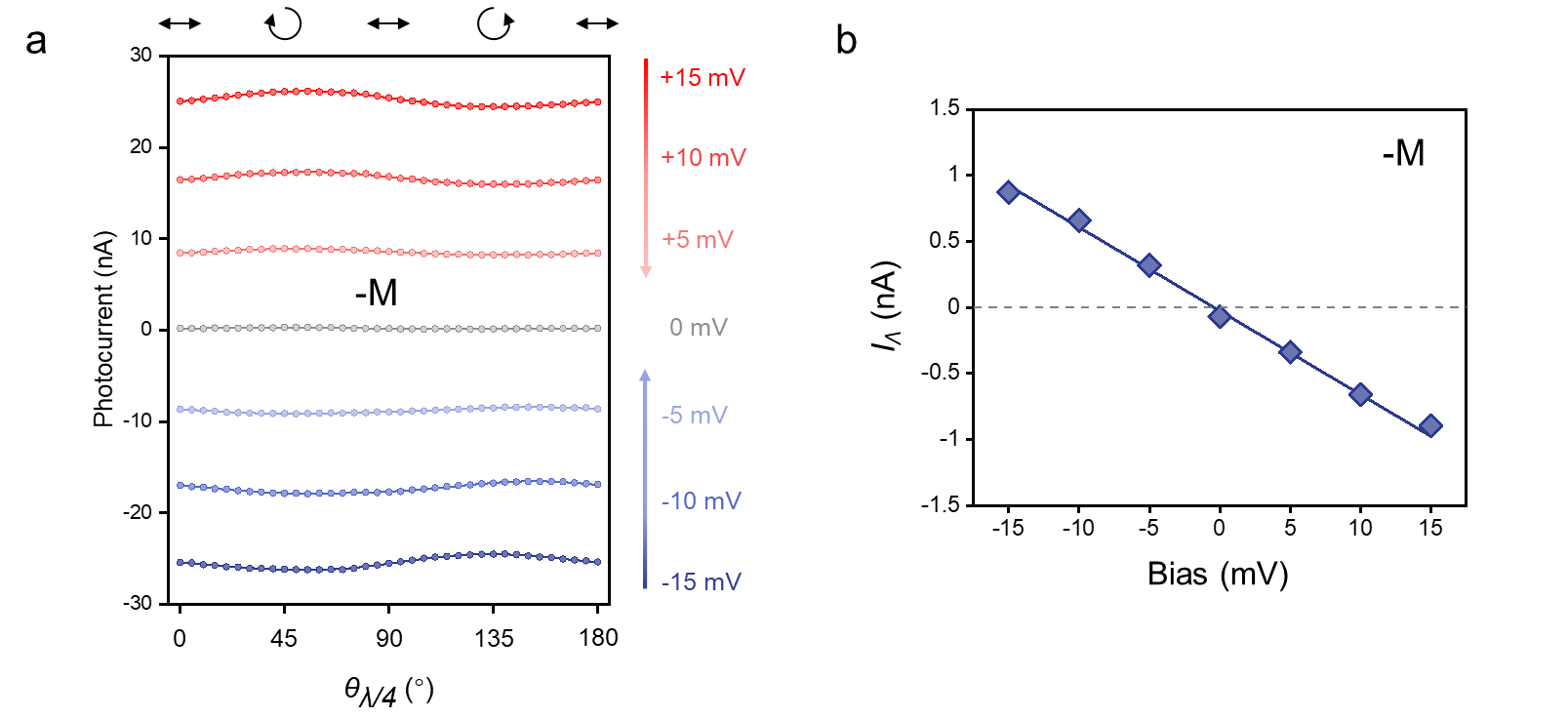


**Figure S13.** Bias dependence of the light chirality-dependent photocurrent under 10.6-μm excitation at 140 K. (a) Photocurrent measured at different QWP angles under several specific biases. (b) *I_Λ_* measured under 10.6-μm excitation as a function of external bias, which are extracted from (a). The measurements were performed on Co_3_Sn_2_S_2_ device 1 after cooling to 140 K with -M magnetization.

In addition, we also performed measurements at a lower temperature (25 K). The measurements were performed on Co_3_Sn_2_S_2_ device 3 after cooling to 25 K with +*M* magnetization. As shown in Fig. S14a, the light chirality-dependent photocurrent still has a linear dependence on the external electric field at 25 K. For clarity, we selectively plot the photocurrent measured at different QWP angles under several specific biases in Fig. S14b, from which the *I_Λ_* values are extracted.


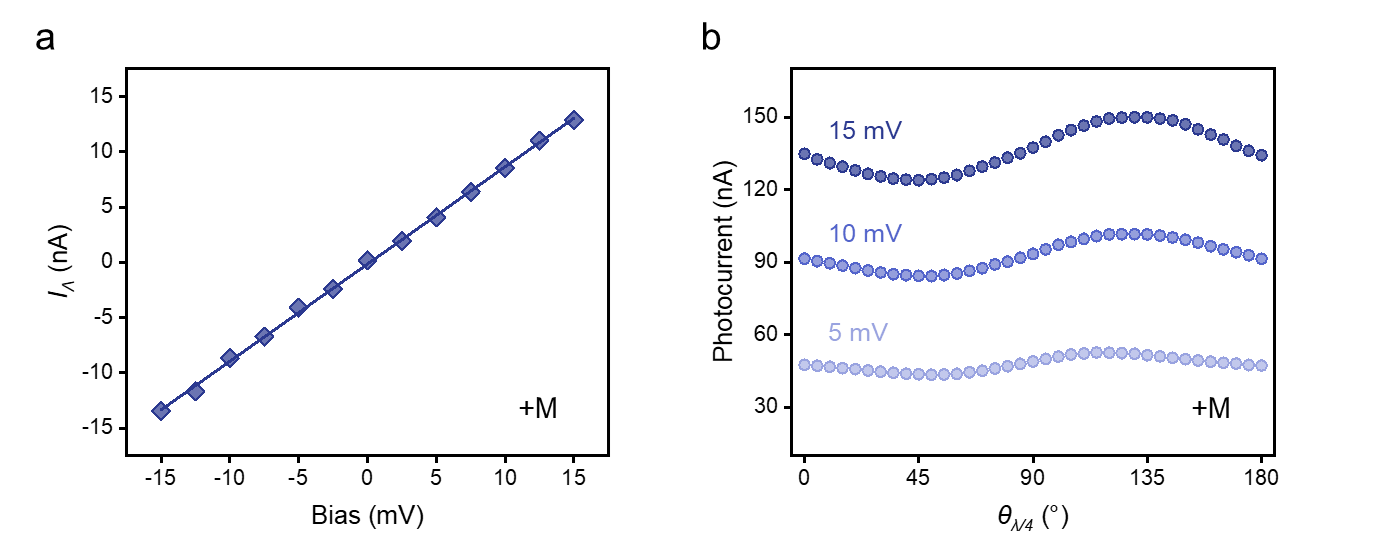


**Figure S14.** Bias dependence of the light chirality-dependent photocurrent under 10.6-μm excitation at 25 K. (a) *I_Λ_* measured under 10.6-μm excitation at 25 K as a function of external bias. (b) Photocurrent measured at different QWP angles under several specific biases. The measurements were performed on Co_3_Sn_2_S_2_ device 3 after cooling to 25 K with +*M* magnetization.

**IX. Optical matrix element in the vicinity of the Weyl point**

The optical dipole matrix elements for circularly polarized light are calculated as:

$$\begin{aligned} r_{cvk}^{\pm}=r_{cvk}^{x}\pm ir_{cvk}^{y}.\#\left( S1 \right) \end{aligned}$$

Here, $r_{cvk}^{x,y}$ is the *x, y* component of the Berry connection at the *k* point between topological bands related to the Weyl point (denoted as $c$ for the conduction band and $v$ for the valence band), “$+$” is for LCP light, and “$-$” is for RCP light. The Berry connection $\boldsymbol{r}_{cvk}$ is calculated from the Hamiltonian and position operator extracted from the output of the Wannier90 package [1] combined with the Quantum Espresso package [2] employing Perdew–Burke–Ernzerhof exchange and correlation functional and pseudopotential methods [3]. As shown in Fig. S15, the optical dipole matrix element is larger for RCP excitation than for LCP excitation on the left side of the Weyl point, whereas it is larger for LCP excitation than for RCP excitation on the right side, which is consistent with the optical selection rule shown in Fig. 1a of the maintext.


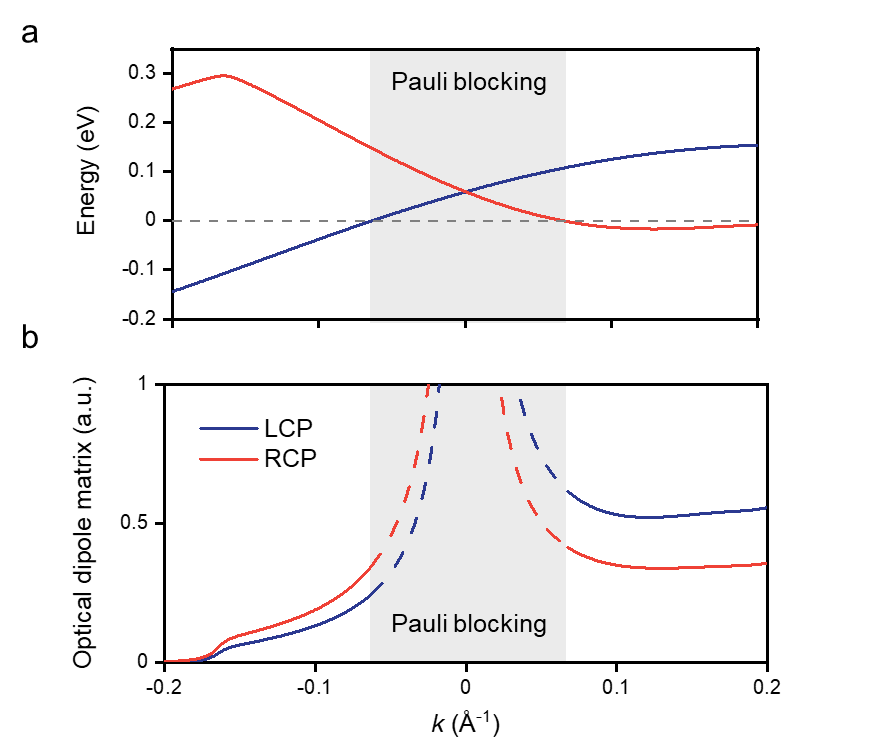


**Figure S15.** Optical matrix element in the vicinity of the Weyl point. (a) Band structure along the direction of the line connecting a pair of Weyl points. The figure only shows the topological bands related to the Weyl point for clarity, which are marked in red and blue. (b) Optical dipole matrix elements for interband transitions along the momentum cut in (a). The blue and red curves denote the results under left- and right-circularly polarized excitation, respectively. The gray region represents the area where optical transitions are forbidden due to Pauli blocking.

**X. Potential contributions to the third-order nonlinear photocurrent**

In this section, we discuss the potential contributions to the third-order nonlinear photocurrent. With the application of a DC electric field in Co_3_Sn_2_S_2_, the generation of a photocurrent is a third-order nonlinear optical response. Considering the DC electric field $E_{dc}$ and the optical fields $E_{\omega}$ and $E_{-\omega}$, the microscopic mechanism of the photocurrent can be understood by acting on electrons successively and can be divided into three types of processes:

(1) The electric fields are in a sequence of $E_{dc}\to E_{\omega}\to E_{-\omega}$, and $E_{dc}\to E_{-\omega}\to E_{\omega}$. This sequence corresponds to the physical process in which the DC electric field first modifies the electronic states in both the band structure through interband coupling [4] and the Fermi surface tilt through intraband coupling [5], and it breaks the inversion symmetry of Co_3_Sn_2_S_2_. Then, the optical field generates a photocurrent, as is the case in an inversion symmetry broken system. This process is discussed in Figs. 3h and 3i of the main text.

(2) The electric fields are in a sequence of $E_{\omega}\to E_{dc}\to E_{-\omega}$, and $E_{-\omega}\to E_{dc}\to E_{\omega}$. This sequence corresponds to a physical process that includes only one intraband motion-induced divergence, and the contribution to the photocurrent is negligible [6]. Therefore, we do not discuss this process in our work.

(3) The electric fields are in a sequence of $E_{\omega}\to E_{-\omega}\to E_{dc}$ and $E_{-\omega}\to E_{\omega}\to E_{dc}$. This sequence corresponds to the physical process in which the optical field first injects electron‒hole pairs in the conduction and valence bands, after which the DC electric field drives them to generate photocurrents. This process is related to magnetic circular dichroism (MCD).

To illustrate the relationship between process (3) and MCD, we note that for process (3), the photocurrent generation can also be understood through a physical and simple three-step model: first, under the excitation of continuous wave light, extra carrier populations are generated by the absorption of photons through interband transitions; second, scattering processes (such as carrier‒carrier interactions and electron‒phonon interactions) relax the energy of the excited carriers and redistribute them to the Fermi surface; and third, by applying a DC electric field, these redistributed carriers are driven to generate current, i.e., the photocurrent. This model only considers the carrier population (or carrier density) dependence of the current, and optical excitation changes the carrier population and subsequently the current; thus, the excited carrier population is proportional to the absorption. In this diagram, we can simply attribute the observed *I_Λ_* to the different absorption coefficients of LCP and RCP light in the FM phase, which is usually referred to as MCD [7]; then, the light chirality-dependent photocurrent *I_Λ_* is directly related to the MCD.

**XI. Numerical calculations of magnetic circular dichroism**

In this section, we present numerical calculations of magnetic circular dichroism (MCD). In the normal incidence configuration, the optical response of Co_3_Sn_2_S_2_ is determined by the in-plane components of the optical conductivity tensor. According to symmetry considerations [8], the in-plane components of the optical conductivity tensor $\boldsymbol{\sigma}$ can be expressed as:

$\begin{aligned} \boldsymbol{\sigma}=\left( \begin{matrix} \sigma_{xx} & \sigma_{xy} \\ -\sigma_{xy} & \sigma_{xx} \end{matrix} \right)\boldsymbol{\#}\left( S2 \right) \end{aligned}$

For circularly polarized light with a polarization vector $\boldsymbol{E}_{s}=\frac{{\hat{\boldsymbol{e}}}_{x}+is {\hat{\boldsymbol{e}}}_{y}}{\sqrt{2}}$ with $s=\pm$ for LCP ($+$) and RCP ($-$) light, the optical current can be expressed as $\boldsymbol{J}_{\boldsymbol{s}}=\epsilon_{0}\sigma_{s}\boldsymbol{E}_{\boldsymbol{s}}$ with:

$\begin{aligned} \sigma_{s}=\sigma_{xx}+is\sigma_{xy}.\#\left( S3 \right) \end{aligned}$

Therefore, the LCP and RCP light give rise to two eigenmodes of light with different susceptibilities. Correspondingly, the refractive index for circularly polarized light can then be calculated as:

$$\begin{aligned} n_{s}^{2}=1+\frac{\sigma_{s}}{-i\tilde{\omega} c\epsilon_{0}}=1+\frac{\sigma_{xx}+is\sigma_{xy}}{-i\tilde{\omega} c\epsilon_{0}},\#\left( S4 \right) \end{aligned}$$

where $\tilde{\omega}=\frac{\omega}{c}$ is the wavevector in vacuum and where $\omega$ is the photon angular frequency.


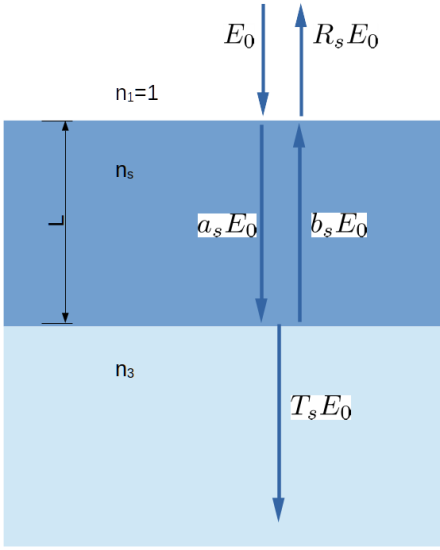


**Figure S16.** Illustration of the sample structure of vacuum/Co_3_Sn_2_S_2_/sapphire and the field amplitudes for upward/downward propagating light in each layer. The top layer is vacuum, the refractive index is $n_{1}=1$, the field amplitudes are $E_{0}$ and $R_{s}E_{0}$ for the downward and upward-propagating lights, respectively; the middle layer is Co_3_Sn_2_S_2_ film with a thickness of *L*, the refractive index is $n_{s}$ for the s-circularly polarized light, the field amplitudes are $a_{s}E_{0}$ and $b_{s}E_{0}$ for the downward and upward propagating lights, respectively; and the substrate is sapphire with a refractive index $n_{3}$ and the amplitude for the transmitted light is $T_{s}E_{0}$.

To calculate how much circularly polarized light is absorbed to excite carriers, we use the transfer matrix method to obtain the electromagnetic field inside the Co_3_Sn_2_S_2_ sample. The structure of the sample is a vacuum/Co_3_Sn_2_S_2_ film/sapphire substrate, as shown in Fig. S16. For $s$-circularly polarized incident light with an amplitude of $E_{0}$, all fields in each layer are still circularly polarized light; then, their amplitudes are $E_{1}\left( z,\omega\right)$ for vacuum, $E_{s}\left( z,\omega\right)$ for the Co_3_Sn_2_S_2_ film layer, and $E_{3}\left( z,\omega\right)$ for the substrate layer, which can be expressed as

$$\begin{aligned} E_{1}\left( z,\omega\right)=E_{0} e^{-i n_{1}\tilde{\omega}z}+R_{s} E_{0} e^{i n_{1}\tilde{\omega}z},\#\left( S5 \right) \end{aligned}$$

$$\begin{aligned} E_{s}\left( z,\omega\right)={a_{s} E}_{0} e^{-i n_{s}\tilde{\omega}z}+b_{s} E_{0} e^{i n_{s}\tilde{\omega}z},\#\left( S6 \right) \end{aligned}$$

$$\begin{aligned} E_{3}\left( z,\omega\right)={T_{s} E}_{0} e^{-i n_{3}\tilde{\omega}z}.\#\left( S7 \right) \end{aligned}$$

Here, *L* is the film thickness, and the refractive indices are $n_{1}=1$ for the vacuum and $n_{3}$ for the sapphire substrate [9]. From the transfer matrix method, the coefficients are as follows:

$$\begin{aligned} a_{s}=\frac{t_{1s}}{1+r_{1s}r_{s3}e^{2in_{s}\tilde{\omega}L}}, b_{s}=r_{s3}a_{s}e^{2in_{s}\tilde{\omega}L}, \#\left( S8 \right) \end{aligned}$$

$$\begin{aligned} T_{s}=t_{s3}a_{s} e^{in_{s}\tilde{\omega}L}, R_{s}=\frac{r_{1s}+r_{s3}e^{2in_{s}\tilde{\omega}L}}{1+r_{1s}r_{s3}e^{2i n_{s}\tilde{\omega}L}}.\#\left( S9 \right) \end{aligned}$$

with the interface Fresnel coefficients $r_{ij}$ and $t_{ij}$ for $i,j=1,s,3$ as:

$$\begin{aligned} r_{\mathrm{ij}}=\frac{n_{i}-n_{j}}{n_{i}+n_{j}}, t_{ij}=\frac{2n_{i}}{n_{i}+n_{j}}.\#\left( S10 \right) \end{aligned}$$

Now, we can calculate the absorbed energy of the circularly polarized incident light. According to the law of energy conservation, the absorbed light energy in the material is converted to Joule heat, which can be expressed as:

$$\begin{aligned} \left\langle\boldsymbol{j}\left( z,t \right)\cdot\boldsymbol{E}_{s}\left( z,t \right) \right\rangle=2 \mathrm{Re}\left[ \sigma_{s} \right]\left| E_{s}\left( z,\omega\right) \right|^{2},\#\left( S11 \right) \end{aligned}$$

where the current density is calculated as $\boldsymbol{j}\left( z,t \right)=\sigma_{s} E_{s}\left( z,\omega\right)e^{-i\omega t}+c.c.$, the field in the time domain is $\boldsymbol{E}_{s}\left( z,t \right)=\boldsymbol{E}_{s}\left( z,\omega\right)e^{-i\omega t}+c.c.$, and $\langle\cdots\rangle$ indicates a time average over a period. After substituting Equation (S6), we obtain the absorption coefficient $\alpha_{s}\left( z \right)=\frac{\left\langle\boldsymbol{j}\left( z,t \right)\cdot\boldsymbol{E}_{s}\left( z,t \right) \right\rangle}{2c\epsilon_{0}\left| E_{0} \right|^{2}}$ for the $s$-circularly polarized light at position *z* as:

$$\begin{aligned} \alpha_{s}\left( z \right)=\frac{\mathrm{Re}\left[ \sigma_{s} \right]}{c\epsilon_{0}}\left( \left| a_{s} \right|^{2}e^{2 \mathrm{Im}\left[ n_{s} \right]\tilde{\omega}z}+\left| b_{s} \right|^{2}e^{-2 \mathrm{Im}\left[ n_{s} \right]\tilde{\omega}z}+2\mathrm{Re}\left[ b_{s}a_{s}^{*}e^{2 i \mathrm{Re}\left[ n_{s} \right]\tilde{\omega}z} \right] \right).\#\left( S12 \right) \end{aligned}$$

The total absorption parameter $\alpha_{s}=\int_{-L}^{0} \alpha_{s}\left( z \right) dz$ is obtained as:

$$\alpha_{s}=\frac{\mathrm{Re}\left[ \sigma_{s} \right]}{2c\epsilon_{0}\tilde{\omega}}\left| a_{s} \right|^{2}\times$$

$$\begin{aligned} \left( \left( 1+\left| r_{s3} \right|^{2}e^{-2\mathrm{Im}\left[ n_{s} \right]\tilde{\omega}L} \right)\frac{1-e^{-2 \mathrm{Im}\left[ n_{s} \right]\tilde{\omega}L}}{\mathrm{Im}\left[ n_{s} \right]}+2e^{-2\mathrm{Im}\left[ n_{s} \right]\tilde{\omega}L}\mathrm{Im}\left[ r_{s3}\frac{e^{2 i \mathrm{Re}\left[ n_{s} \right]\tilde{\omega}L}-1}{\mathrm{Re}\left[ n_{s} \right]} \right] \right). \#\left( S13 \right) \end{aligned}$$

Therefore, the required material parameter for the calculation of the MCD is the optical conductivity $\sigma_{s}$ of Co_3_Sn_2_S_2_. In the calculation, we utilized the experimentally obtained optical conductivity of Co_3_Sn_2_S_2_ [10]. Experimentally, the optical conductivities $\sigma_{xx}$ and $\sigma_{xy}$ are extracted from the magneto-optical response [10], and they are plotted as solid curves in Fig. S17a. The solid curves in Fig. S17b show the corresponding $\sigma_{\pm}$ values from $\sigma_{xx}$ and $\sigma_{xy}$ in Fig. S17a. After obtaining the above parameters, the MCD is then calculated as $\frac{2(\alpha_{-}-\alpha_{+})}{\alpha_{-}+\alpha_{+}}$ and the result is shown in Fig. 3j of the maintext.


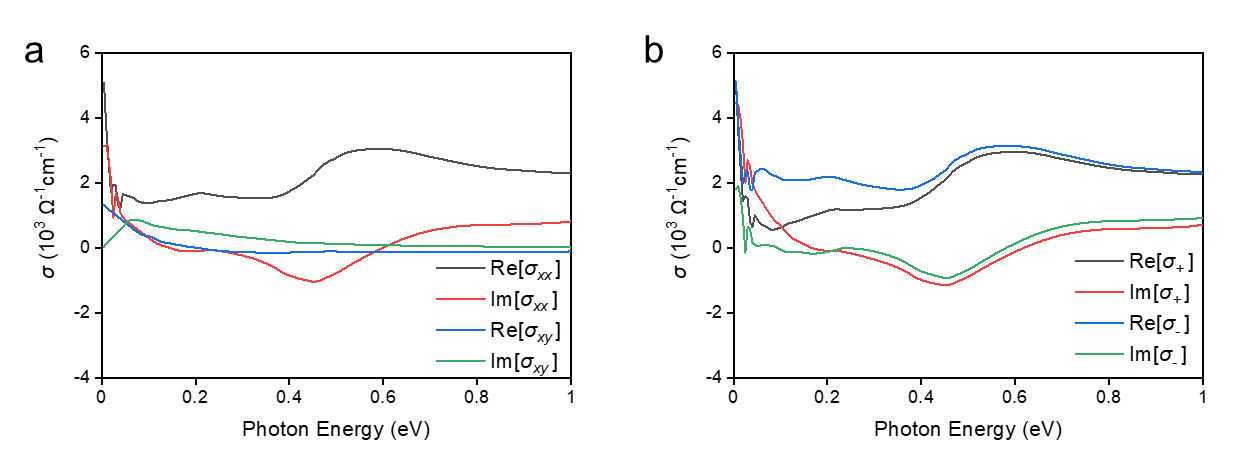


**Figure S17.** Experimental spectra of the optical conductivities. (a) Experimental spectra of the optical conductivities $\sigma_{xx}$ and $\sigma_{xy}$. The experimental data are extracted from ref. [10]. (b) The corresponding spectra of the optical conductivities $\sigma_{\pm}$, which are calculated from the data plotted in (a).

**XII. Tunability of** ***I_Λ_* measured in the central area of the sample**

In this section, we present the tunability of *I_Λ_* measured at the central area of the sample. The bias dependence of *I_Λ_* measured at the central area of the sample under +*M* magnetization is shown in Fig. S18a. For clarity, we also extracted *I_Λ_* and plotted it as a function of bias voltage, as shown in Fig. S18b.


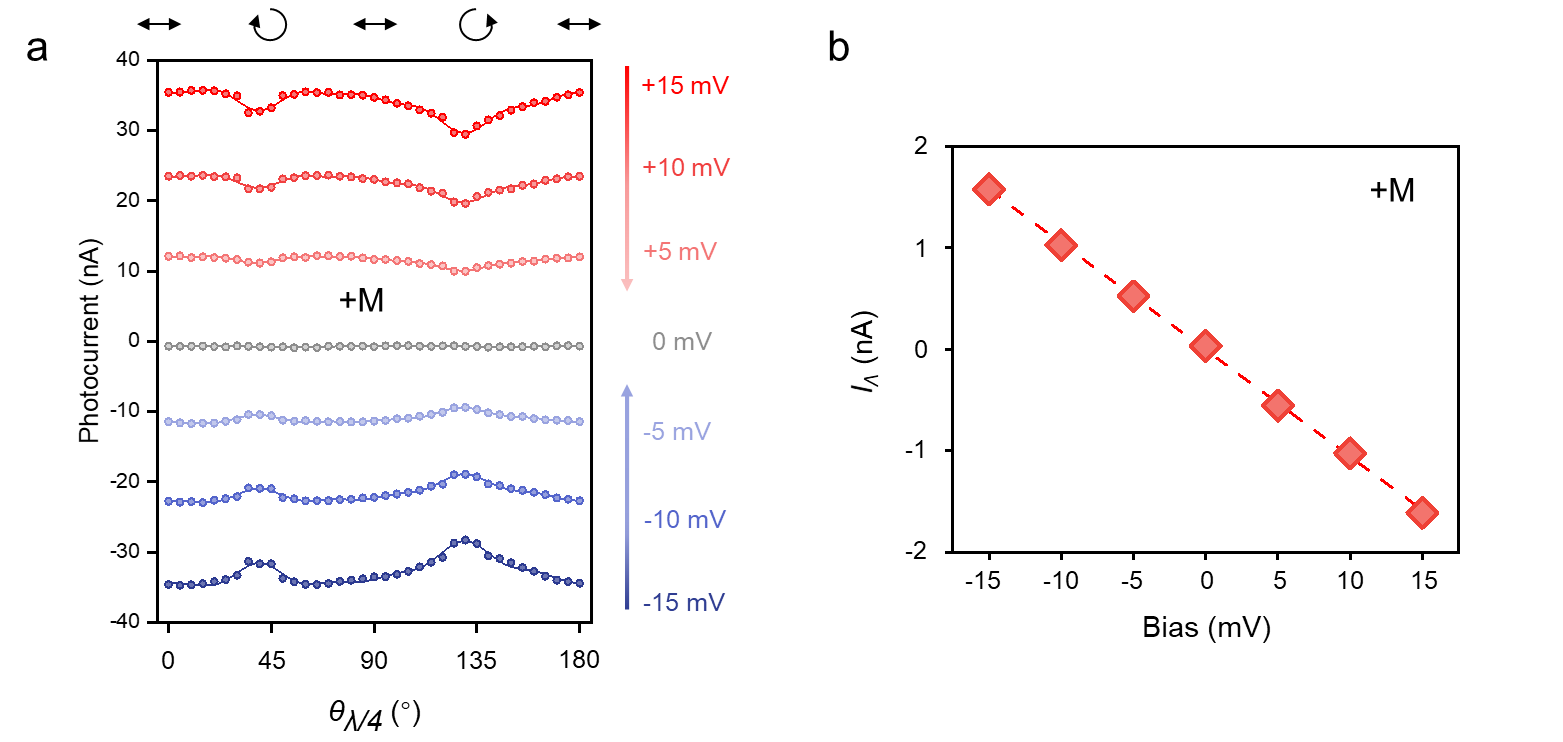


**Figure S****18.** Tunability of *I_Λ_* measured at the central area of the sample. (a) Measurements performed under +*M* magnetization. The excitation power was 1.5 mW. (b) Dependence of *I_Λ_* on the external bias under +*M* magnetizations.

**References**

1. Pizzi G, Vitale V, Arita R *et al.* Wannier90 as a community code: new features and applications. *J Phys Condens Matter*. 2020; **32**(16): 165902. doi: 10.1088/1361-648X/ab51ff

2. Giannozzi P, Baroni S, Bonini N *et al.* QUANTUM ESPRESSO: a modular and open-source software project for quantum simulations of materials. *J Phys Condens Matter*. 2009; **21**(39): 395502. doi: 10.1088/0953-8984/21/39/395502

3. Perdew JP, Burke K, Ernzerhof M. Generalized Gradient Approximation Made Simple. *Phys Rev Lett*. 1996; **77**(18): 3865-8. doi: 10.1103/PhysRevLett.77.3865

4. Cheng JL, Vermeulen N, Sipe JE. DC current induced second order optical nonlinearity in graphene. *Opt Express*. 2014; **22**(13): 15868-76. doi: 10.1364/OE.22.015868

5. Manini N. Solids. In: Manini N (ed.) *Introduction to the Physics of Matter: Basic Atomic, Molecular, and Solid-State Physics*. Cham: Springer; 2020. 149-244.

6. Cheng JL, Sipe JE, Wu SW *et al.* Intraband divergences in third order optical response of 2D systems. *APL Photonics*. 2019; **4**(3): 034201. doi: 10.1063/1.5053715

7. Stephens PJ. Magnetic Circular Dichroism. *Annu Rev Phys Chem*. 1974; **25**(1): 201-32. doi: 10.1146/annurev.pc.25.100174.001221

8. Boyd RW. In: Boyd RW (ed.) *Nonlinear Optics*. Burlington: Academic Press; 2008. 1-67.

9. Querry MR. *Optical constants*. 1985: Contractor Report CRDC-CR-85034.

10. Okamura Y, Minami S, Kato Y *et al.* Giant magneto-optical responses in magnetic Weyl semimetal Co_3_Sn_2_S_2_. *Nat Commun*. 2020; **11**(1): 4619. doi: 10.1038/s41467-020-18470-0
